# Construction and validation of a nomogram model for predicting the overall survival of colorectal cancer patients

**DOI:** 10.1186/s12893-023-02018-2

**Published:** 2023-06-29

**Authors:** Guo Peiyuan, Hu xuhua, Guo Ganlin, Yin Xu, Liu Zining, Han Jiachao, Yu Bin, Wang Guiying

**Affiliations:** 1 The Second General Surgery, The Fourth Hospital of Hebei Medical University, NO.12, JianKang Road, Shijiazhuang, Hebei Province PR China; 2 The Department of Gastrointestinal Surgery, The Third Hospital of Hebei Medical University, NO.139, Ziqiang Road, Shijiazhuang, Hebei Province PR China; 3 The Department of General Surgery, The Second Hospital of Hebei Medical University, No. 215, Heping West Road, Shijiazhuang, Hebei Province PR China

**Keywords:** Colorectal cancer, Overall survival, Nomogram

## Abstract

**Background:**

Colorectal cancer (CRC) is a frequent cancer worldwide with varied survival outcomes.

**Objective:**

We aimed to develop a nomogram model to predict the overall survival (OS) of CRC patients after surgery.

**Design:**

This is a retrospective study.

**Setting:**

This study was conducted from 2015 to 2016 in a single tertiary center for CRC.

**Patients:**

CRC patients who underwent surgery between 2015 and 2016 were enrolled and randomly assigned into the training (n = 480) and validation (n = 206) groups. The risk score of each subject was calculated based on the nomogram. All participants were categorized into two subgroups according to the median value of the score.

**Main outcome measures:**

The clinical characteristics of all patients were collected, significant prognostic variables were determined by univariate analysis. Least absolute shrinkage and selection operator (LASSO) regression was applied for variable selection. The tuning parameter (λ) for LASSO regression was determined by cross-validation. Independent prognostic variables determined by multivariable analysis were used to establish the nomogram. The predictive capacity of the model was assessed by risk group stratification.

**Results:**

Infiltration depth, macroscopic classification, BRAF, carbohydrate antigen 19 − 9 (CA-199) levels, N stage, M stage, TNM stage, carcinoembryonic antigen levels, number of positive lymph nodes, vascular tumor thrombus, and lymph node metastasis were independent prognostic factors. The nomogram established based on these factors exhibited good discriminatory capacity. The concordance indices for the training and validation groups were 0.796 and 0.786, respectively. The calibration curve suggested favorable agreement between predictions and observations. Moreover, the OS of different risk subgroups was significantly different.

**Limitations:**

The limitations of this work included small sample size and single-center design. Also, some prognostic factors could not be included due to the retrospective design.

**Conclusions:**

A prognostic nomogram for predicting the OS of CRC patients after surgery was developed, which might be helpful for evaluating the prognosis of CRC patients.

## Introduction

Colorectal cancer (CRC) is the third most diagnosed carcinoma and the leading cause of cancer-related death worldwide. It is the third most common cancer in women and the second in men [1, 2]. Globally, there were over 1 million newly diagnosed cases and approximately 500,000 deaths per year [3]. In China, the incidence and mortality of CRC continue to increase [4, 5].

Surgical intervention remains the main treatment strategy for CRC and adjuvant therapy is recommended for high-risk patients. The TNM staging system is widely applied to predict the prognosis for CRC patients. However, significant heterogeneity in the survival outcomes of patients at the same TNM stage has been observed due to the diversity of cancer biology and clinicopathological characteristics [6, 7]. Moreover, the TNM alone cannot provide enough individualized predictions for postoperative CRC patients [8, 9]. Hence, it is necessary to develop a prognostic model for personalized probabilistic predictions.

Nomograms are statistical tools that calculate the probability of individual clinical events based on determinants and prognostic characteristics [10]. Nomogram prediction models provide individualized probability estimates of death for each patient. Several studies have established nomogram models to predict overall postoperative survival of colorectal cancer patients [11–13]. Some variables are included in these nomogram models, such as age and sex. However, it is worth noting that these studies are characterized by long time span of data sets and few variables included. New pathologic and molecular markers such as perineuronal infiltration, mismatch repair status, and RAS/RAF mutation status were not included in the data analysis. As a result, these columns are not applicable to the current patient. In this study, these new pathological and molecular markers were also incorporated into the data analysis. This will help predict survival rates for colon cancer patients more accurately.

In this study, a prognostic nomogram model for personalized probabilistic predictions of the overall survival (OS) of CRC patients after surgery was developed using tumor-related factors and patient-related factors (e.g. age, diabetes, hypertension). This model may help physicians predict the OS of each patient without incurring additional costs.

## Materials and methods

### Subjects and ethical approval

**Fig. 1:**
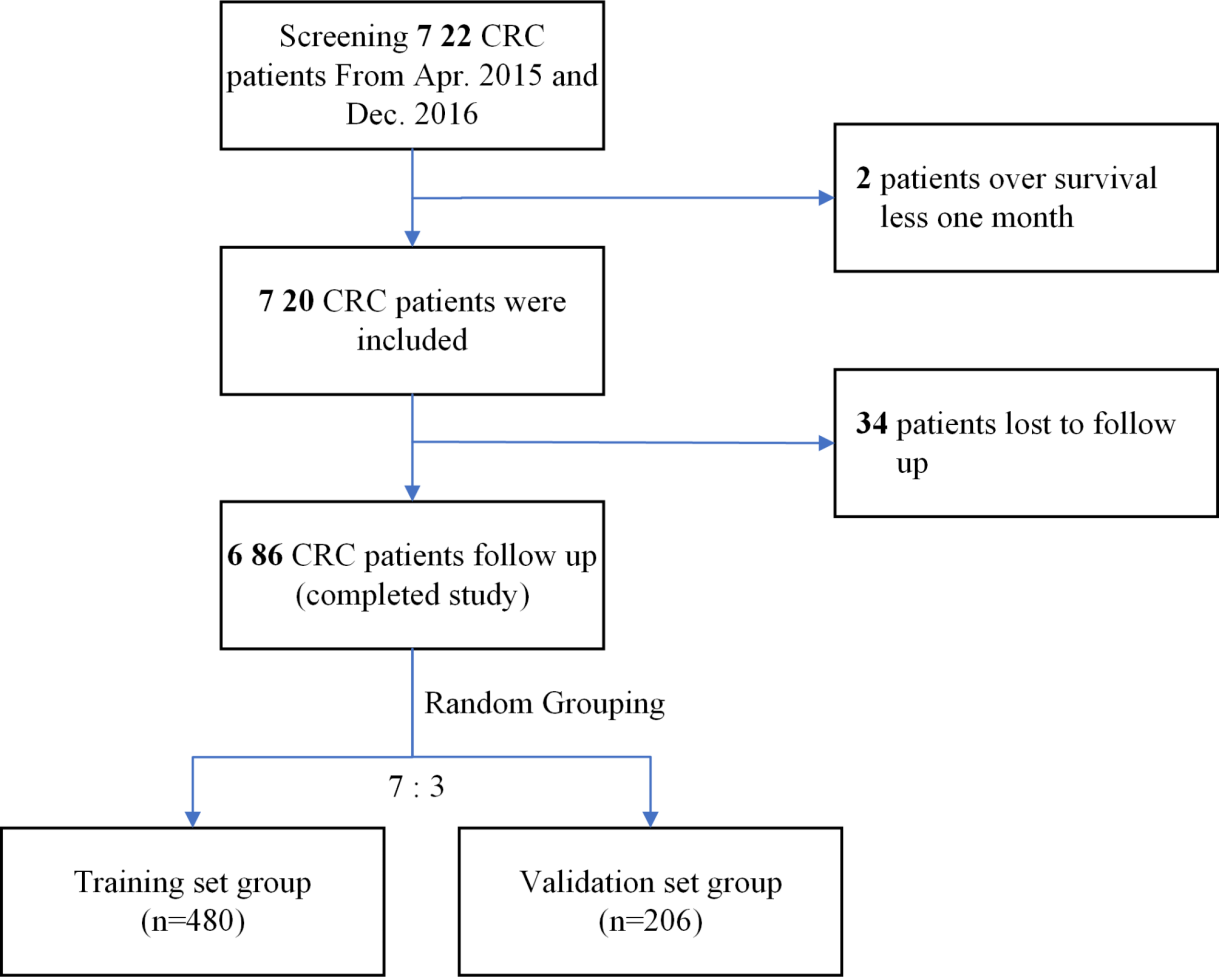
The Research flow chart for this study

In this retrospective, single-center study, CRC patients who underwent surgery in our hospital between April 2015 and December 2016. The inclusion criteria: (1) the diagnosis of CRC was made in our department and confirmed by pathological findings; (2) patients underwent radical surgical excision to remove primary tumors. The following cases were excluded: (1) patients with unknown prognosis or incomplete follow-up data; (2) patients who had received radiotherapy, chemotherapy, or chemoradiotherapy prior to enrollment; (3) patients with other primary malignant tumors, acute infection, or severe liver disease; (4) patients who have died from non-cancer causes. Finally, 686 patients were enrolled. The case registration process is shown in Fig. 1. The start of the follow-up was the patient’s diagnosis time, the end of the follow-up was on July 9, 2020, the follow-up duration was 7 to 75 months, and the median follow-up duration was 27.00 months.

This work was registered at China Clinical Trial Registry (Registration No. ChiCTR 2,100,043,775) and approved by local ethics committee (Approval No. 2020kt417). Informed consent was waived because of the retrospective design.

### Clinical data

The clinical characteristics of all patients were collected, including sex, age, BRAF type, KRAS type, MLH1 type, MSH2 type, MSH6 type, PMS2 type, tumor site, history of diabetes, history of hypertension, history of aspirin use, carbohydrate antigen 19 − 9 (CA-199) levels, CA-724 levels, carcinoembryonic antigen (CEA) levels, WBC count (i.e. leukocytes and white blood cells), neutrophil count, platelet count, platelet to lymphocyte ratio (PLR), pathological type, degree of differentiation, infiltration depth, lymphocyte count, neutrophil to lymphocyte ratio (NLR), vascular tumor thrombus, nerve invasion, N stage, M stage, number of positive lymph node (LN), TNM stage, LN metastasis, macroscopic classification, tumor size (long diameter) and Chemotherapy.

### Variable selection

Univariate Cox regression analysis was used to explore the clinical characteristics that associated with the OS of CRC patients significantly. Least absolute shrinkage and selection operator (LASSO) regression is a shrinkage method that can reduce the likelihood of overfitting by actively selecting from a large set of variables and decreasing the regression coefficient [14, 15]. In this study, significant variables identified by univariate analysis were selected by LASSO regression using the R package “glmnet”. Dummy variables were generated for categorical variables. The tuning parameter λ was determined by cross-validation. Finally, multivariate Cox regression analysis was performed to identify independent risk or protective factors for the OS of patients. P < 0.05 indicated statistical significance.

### Nomogram construction

The R package “rms” was used to construct the nomogram model. Independent risk or protective factors were included in the nomogram and 1-, 3-, and 5-year OS were predicted.

### Nomogram validation

The concordance index (C-index; range: 0.5–1.0) was calculated using the R software to validate the nomogram. The closer to 1 the value, the higher the discriminatory capacity of the model. The C-indices were calculated by Cox regression models and the discrimination for nomogram models were estimated by C-index. Calibration curves were used to assess the difference between actual observations and predictions by the nomogram, in which a 45-degree line indicated perfect agreement between observed and predicted probabilities. C-index is between 0.5 and 1. 0.5 is completely random, indicating that the model has no predictive effect, and 1 is completely consistent, indicating that the prediction results of the model are completely consistent with reality. Previous studies believed that C-index between 0.50 and 0.70 was low accuracy; between 0.71 and 0.90 was moderate accuracy; and higher than 0.90 was high accuracy [16–18]. Decision curve analysis (DCA) is a widely used method to measure clinical utility [19, 20]. In this study, the threshold probability and net benefit of the nomogram and TNM stage were determined by DCA using the R package “rmda”. The sensitivity and specificity of nomogram was determined by receiver operating characteristics (ROC) curves.

### Risk group stratification

The risk score of each subject was calculated using the formula:

1 $${\rm{Risk score = }}\sum _{i = 1}^n{\rm{ORi}}*{\rm{xi}}$$

The OR_i_ was the OR value in multivariate Cox regression analysis and x_i_ was the z-score-transformed value of each factor in nomogram. All participants were categorized into high-risk and low-risk group according to the median value of the score. Score distribution in all patients and the number of patients with low or high scores were assessed.

### Statistical analysis

R software (version 4.0.1) and SPSS (version 20.0; Chicago, IL, USA) were used for data analysis. Independent t test or Mann-Whitney U test were used for continuous variables, and Fisher’s exact test or Chi-square test were used for categorical variables to evaluate the differences in clinical features among each group. Three or more groups were compared using Analysis of variance (ANOVA) and Kruskal-Wallis H test. Survival curves were generated by Kaplan–Meier method and compared by two-sided log-rank test. P < 0.05 indicated statistical significance.

## Results

### Patients

A total of 686 CRC patients (403 males; 283 females) were enrolled and randomly assigned into two groups at a ratio of 7:3 (training group, n = 480; validation group, n = 206). The clinical characteristics of both groups are shown in Table 1.

**Table 1:** The baseline characteristics of training and validations sets

Clinical variables	Training set	Validation set	*P*-value
Total	480	206	
Sex (%)			0.9976
Male	282 (58.8)	121 (58.7)	
Female	198 (41.2)	85 (41.3)	
Age (years)			
Median (IQR)	59 (51–64)	57.5 (50–64)	0.2370
BRAF			0.3389
Wild type	464 (96.7)	196 (95.1)	
Mutant type	16 (3.3)	10 (4.9)	
KRAS			0.2605
Wild type	288 (60.0)	133 (64.6)	
Mutant type	192 (40.0)	73 (35.4)	
MLH1			0.9204
Wild type	444 (92.5)	191 (92.7)	
Mutant type	36 (7.5)	15 (7.3)	
MSH2			0.7817
Wild type	459 (95.6)	196 (95.1)	
Mutant type	21 (4.4)	10 (4.9)	
MSH6			0.9967
Wild type	452 (94.2)	194 (94.2)	
Mutant type	28 (5.8)	12 (5.8)	
PMS2			0.7898
Wild type	441 (91.9)	188 (91.3)	
Mutant type	39 (8.1)	18 (8.7)	
Tumor site			0.8266
Ascending colon	96 (20.0)	34 (16.5)	
Transverse colon	9 (1.9)	4 (1.9)	
Descending colon	26 (5.4)	12 (5.8)	
Sigmoid colon	89 (18.5)	36 (17.5)	
Rectum	260 (54.1)	120 (58.2)	
Diabetes			0.3733
No	422 (87.9)	176 (85.4)	
Yes	58 (12.1)	30 (14.6)	
Hypertension			0.4955
No	341 (71.0)	141 (68.4)	
Yes	139 (29.0)	65 (31.6)	
Aspirin medication history			0.0450
No	438 (91.3)	197 (95.6)	
Yes	42 (8.7)	9 (4.4)	
CEA			0.8270
Median (IQR)	3.53 (2.03-7.8875)	3.475 (1.87-8.7225)	
CA-199			0.1690
Median (IQR)	13.01 (7.8975–23.585)	15.23 (8.355-23.9375)	
CA-724			0.1510
Median (IQR)	1.2975 (2.64-5.6225)	2 (1.115–5.0375)	
WBC			0.5930
Median (IQR)	7.4 (5.82-10.1625)	7.175 (5.6075–9.7325)	
Neutrophils			
Median (IQR)	5 (3.6-8.1425)	4.64 (3.5-7.6725)	0.3320
Lymphocytes			0.2110
Median (IQR)	1.4 (0.83–1.86)	1.49 (0.96-1.9275)	
Platelets			0.6530
Median (IQR)	234 (188–290)	230 (187.25–288.5)	
PLR			0.7390
Median (IQR)	48.35 (26.37–71.59)	49.60 (30.89–68.86)	
NLR			0.1790
Median (IQR)	0.31 (0.11–0.46)	0.34 (0.15–0.49)	
Pathological type			0.1113
Adenocarcinoma	372 (77.5)	173 (84.0)	
Mucinous adenocarcinoma	107 (22.3)	32 (15.5)	
Signet ring cell carcinoma	1 (0.2)	1 (0.5)	
Degree of differentiation			0.3128
High differentiation	1 (0.2)	0 (0.0)	
Medium differentiation	348 (72.5)	160 (77.7)	
Low differentiation	131 (27.3)	46 (22.3)	
Infiltration depth			0.7707
1	13 (2.7)	4 (1.9)	
2	52 (10.8)	25 (12.1)	
3	118 (24.6)	45 (21.8)	
4	297 (61.9)	132 (64.1)	
Vascular tumor thrombus			0.4170
No	421 (87.7)	176 (85.4)	
Yes	59 (12.3)	30 (14.6)	
Nerve invasion			0.6466
No	377 (78.5)	165 (80.1)	
Yes	103 (21.5)	41 (19.9)	
Lymph node metastasis			0.9211
No	301 (62.7)	130 (63.1)	
Yes	179 (37.3)	76 (36.9)	
N stage			0.9756
0	301 (62.7)	131 (63.6)	
1	134 (27.9)	56 (27.2)	
2	45 (9.4)	19 (9.2)	
Number of positive Lymph node			0.9830
Median (IQR)	0 (0–1)	0 (0–1)	
Metastasis			0.4989
No	453 (94.3)	197 (95.6)	
Yes	27 (5.6)	9 (4.4)	
TNM stage			0.9100
1	51 (10.6)	23 (11.2)	
2	240 (50.0)	102 (49.5)	
3	162 (33.8)	72 (35.0)	
4	27 (5.6)	9 (4.4)	
Macroscopic classification			0.9249
Ulcer	432 (90.0)	184 (87.6)	
Infiltration	3 (0.6)	1 (0.4)	
Bulge	45 (9.4)	21 (10.2)	
Tumor size (long diameter)			
Median (IQR)	4.5 (3.5–5.5)	4.35 (3.125-5)	
Chemotherapy			0.4828
No	219	88	
Yes	261	118	

### Variable selection

**Fig. 2:**
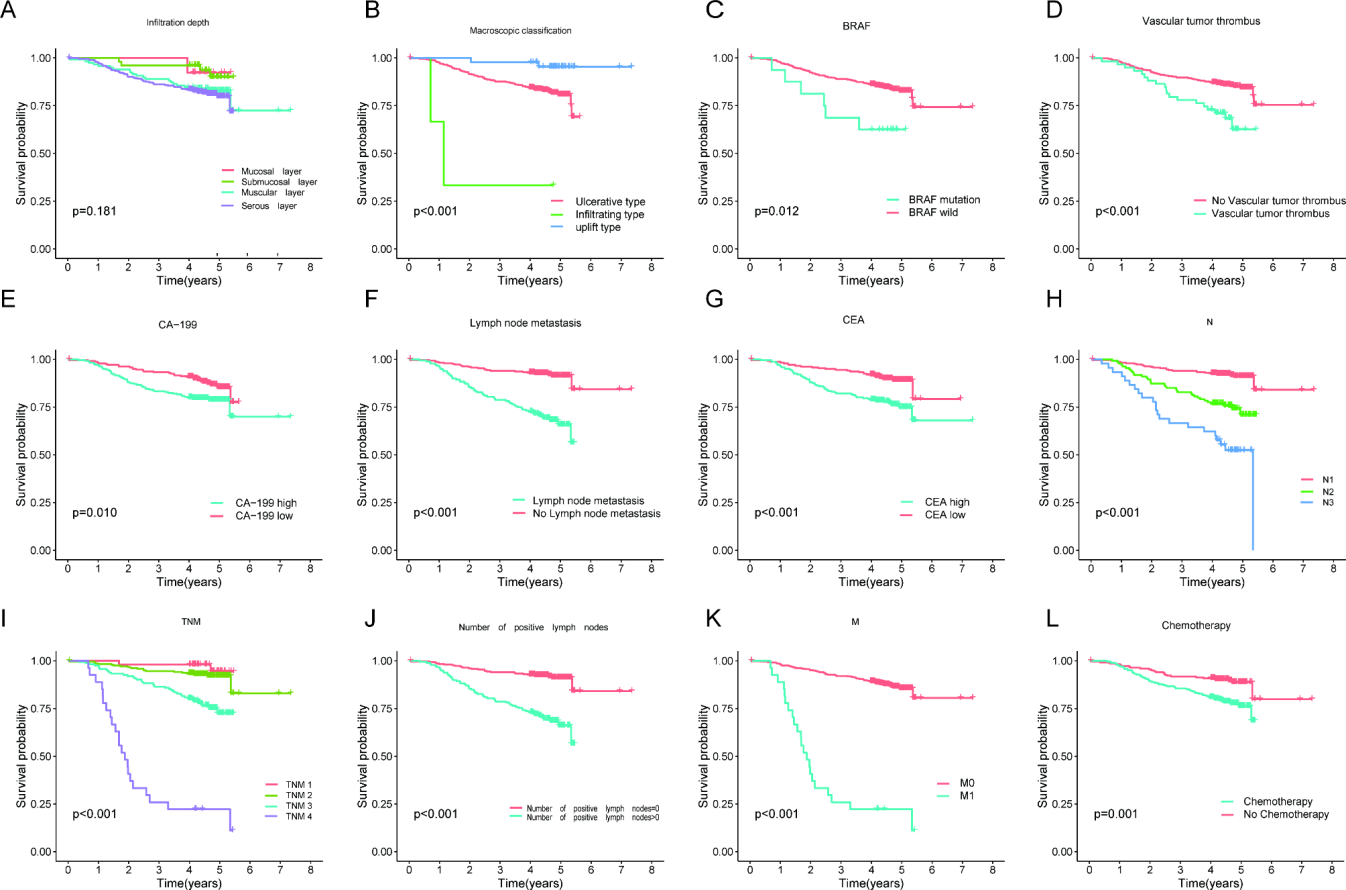
The survival curves of 12 variables had P values less than 0.05. (A) Infiltration depth (B) Macroscopic classification (C) BRAF (D) Vascular tumor thrombus (E) CA-199 (F) Lymph node metastasis (G) CEA (H) N stage (I) TNM stage (J) Number of positive lymph nodes (K) Metastasis (L) Chemotherapy

Univariate Cox regression analysis was used to explore the clinical characteristics that associated with the OS of CRC patients significantly. And in the univariate analysis, 12 variables had P values less than 0.05, which were shown in Table 2. Moreover, the survival curves of 12 variables was shown in Fig. 2.

**Table 2:** Correlative effect on survival of the patient based on Univariate cox analysis

Characteristics	SSI		
	HR	95% CI	P
Infiltration depth	1.401	1.014–1.935	0.041
Macroscopic classification	0.543	0.304–0.972	0.040
BRAF	2.771	1.205–6.371	0.016
Vascular tumor thrombus	2.447	1.462–4.096	0.001
CA-199	1.004	1.003–1.005	< 0.001
Lymph node metastasis	4.399	2.747–7.043	< 0.001
CEA	1.003	1.002–1.004	< 0.001
N stage	2.722	2.069–3.582	< 0.001
TNM stage	4.375	3.169–6.041	< 0.001
Number of positive lymph nodes	1.250	1.192–1.310	< 0.001
Metastasis	12.494	7.545–20.689	< 0.001
Chemotherapy	2.172	1.349–3.496	0.001

OR, odds ratio; 95% CI, 95% confidence interval

**Fig. 3:**
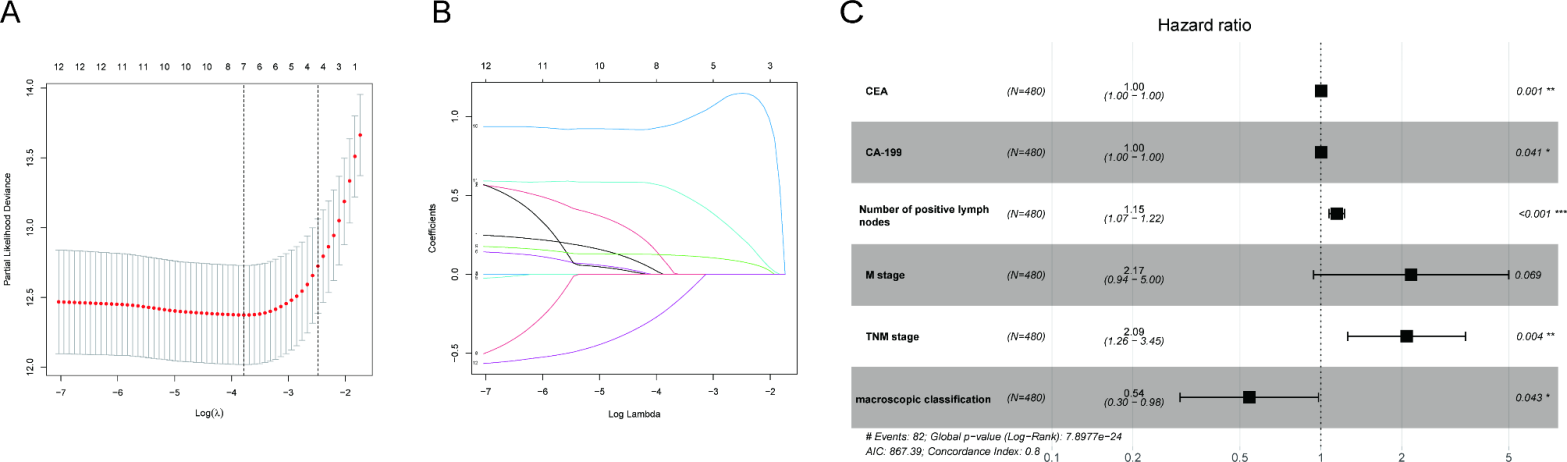
Variables selection using LASSO and multivariate Cox regression analysis. (A, B) Selection of the most appropriate penalty parameter (λ) for LASSO regression, and the LASSO regression selected seven variables. (C). Forrest plot of multivariate Cox regression analysis

The most appropriate tuning parameter (λ) for LASSO regression was 0.12 when partial likelihood binomial deviation reached its minimum value (Fig. 3A). Furthermore, seven characteristics with were selected by LASSO regression, including BRAF, number of positive lymph nodes, CEA levels, CA-199 levels, M stage, TNM stage, and macroscopic classification (Fig. 3B). Multivariate analysis was then applied and five independent risk or protective factors were identified, including CEA levels (OR = 1.00, P = 0.001), CA-199 levels (OR = 1.00, P = 0.041), TNM stage (OR = 2.09, P = 0.004), number of positive LN (OR = 1.15, P < 0.001), and macroscopic classification (OR = 0.54, P = 0.043) (Fig. 3C). The above variables were used to construct the nomogram. As metastasis state is a well-recognized risk factor for the prognosis of CRC patients, M stage was also included in the nomogram model.

### Nomogram construction and performance

**Fig. 4:**
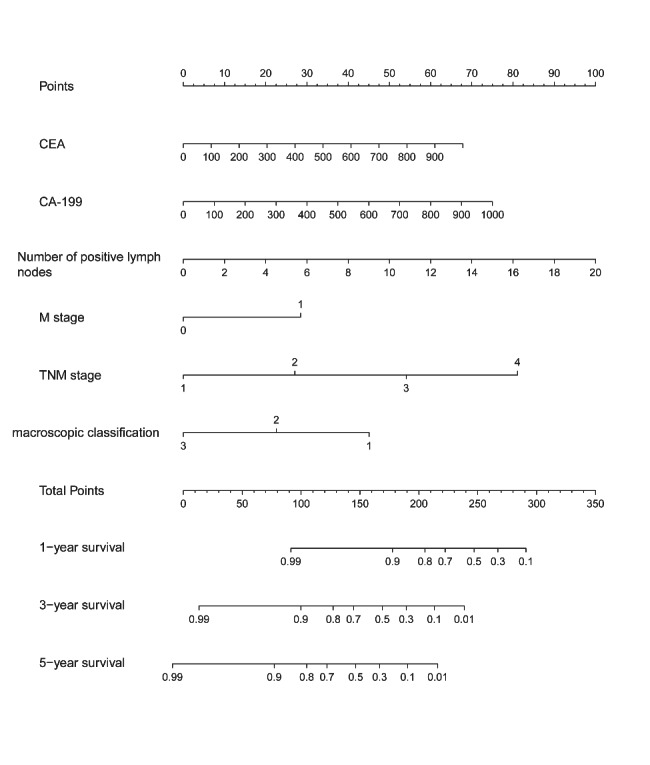
Nomogram model for predicting the survival rate of patients at 1, 3, and 5 years in in the training group

**Fig. 5:**
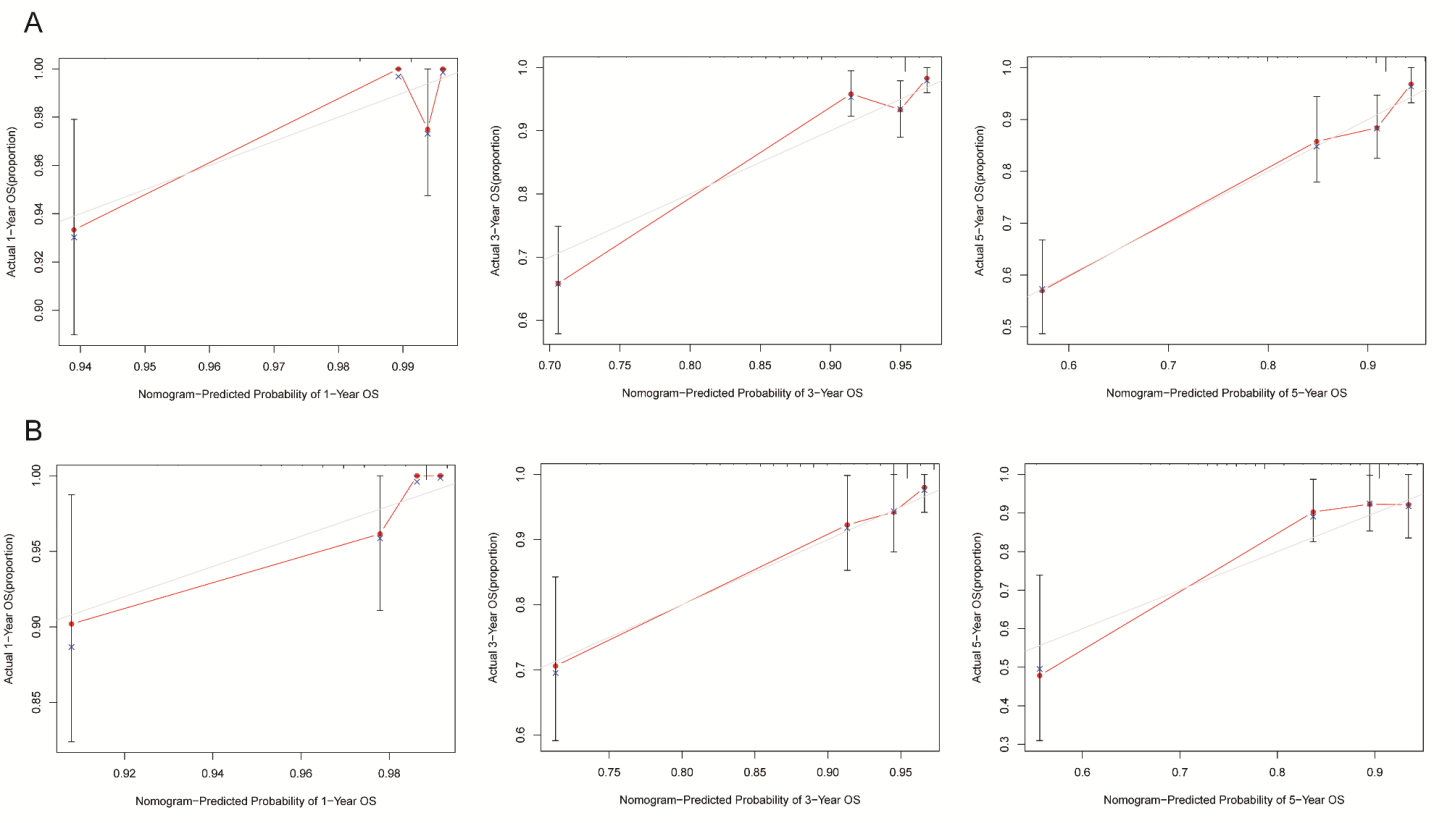
Calibration curves for 1, 3 and 5-year overall survival prediction. (A) The consistency test of the 1-year survival rate of the training group (B) The consistency test of the 3-years survival rate of the training group (C) The consistency test of the 5-year survival rate of the training group. (D) The consistency test of the 1-year survival rate of the validation group (E) The consistency test of the 3-years survival rate of the validation group (F) The consistency test of the 5-years survival rate of the validation group

**Fig. 6:**
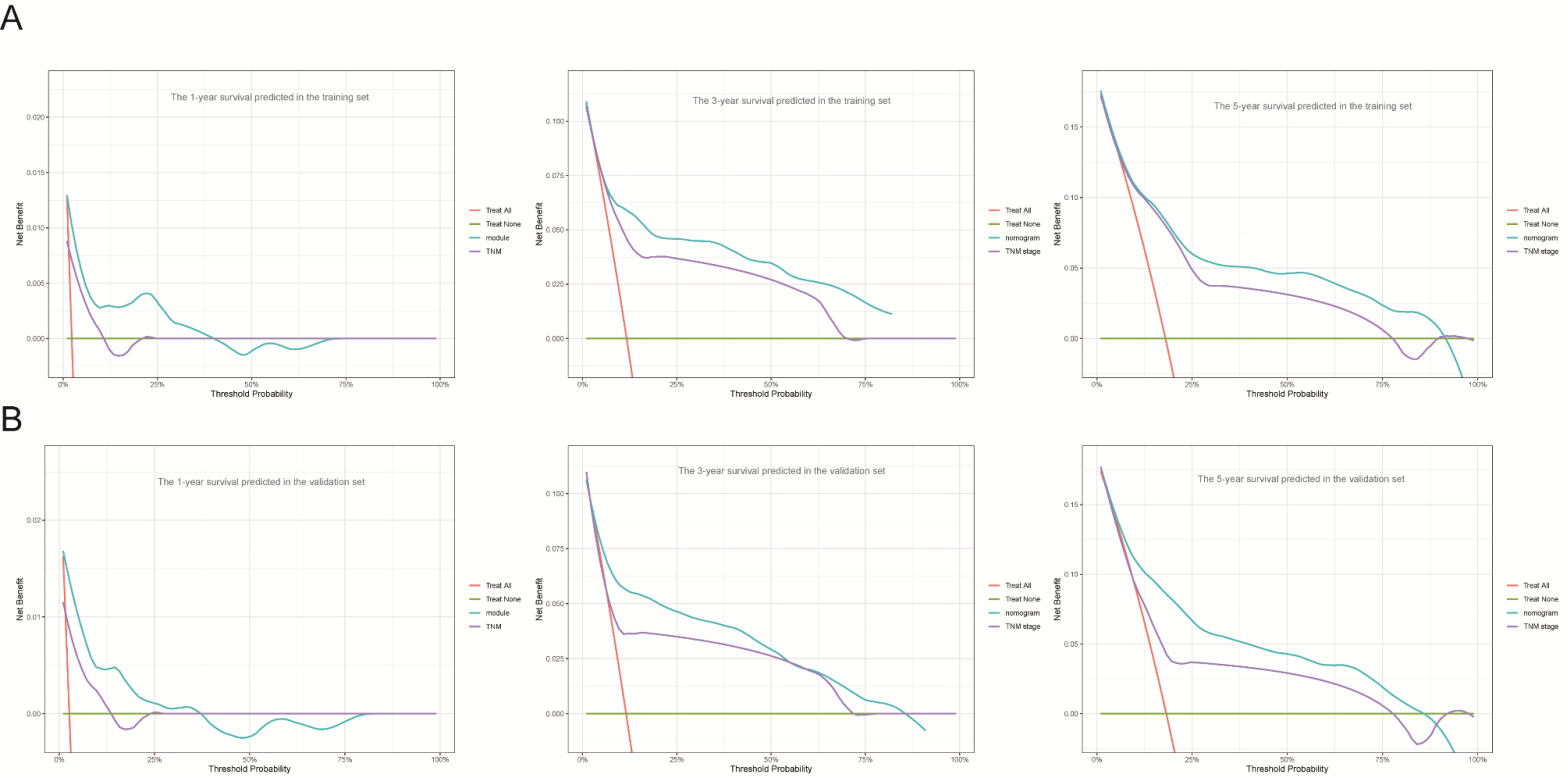
The results of the decision curve analysis (DCA) for nomogram. (A) DCA of 1-year OS using training group. (B) DCA of 3-years OS using training group. (C) DCA of 5-years OS using training group. (D) DCA of 1-year OS using validation group. (E) DCA of 3-years OS using validation group. (F) DCA of 5-years OS using validation group

**Fig. 7:**
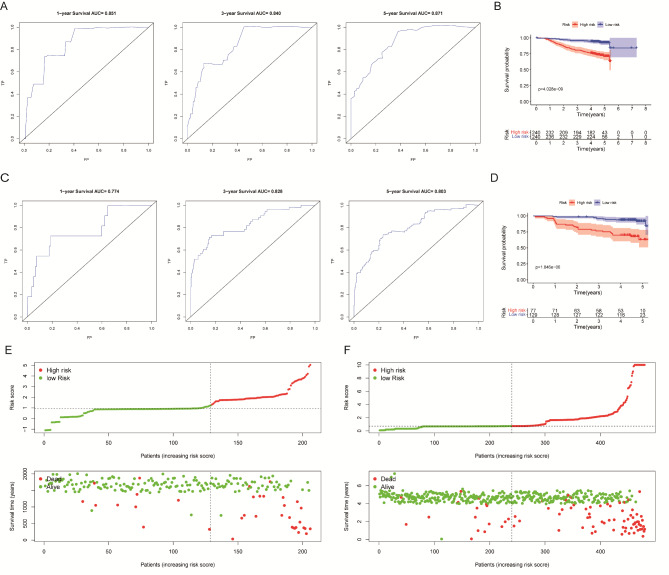
ROC curve and Kaplan-Meier analysis for evaluating the reliability of the prediction model, and distribution of the risk value and survival status. (A) ROC curve of 1、3 and 5-year survival rate predictions on the training group.(B) Kaplan-Meier analysis of the risk value of the training group.(C) ROC curve of 1、3 and 5-year survival rate predictions on the validation group.(D) Kaplan-Meier analysis of the risk value of the validation group.(E)The distribution of the risk value and survival status of the patients in the training group.(F)The distribution of the risk value and survival status of the patients in the validation group

The nomogram was developed using variables selected by multivariate analysis (Fig. 4). A vertical line was drawn from the point on the top row and a point was assigned for each variable. By drawing a vertical line from the points to the result axis, the predicted survival probability can be obtained. For training group, the C-index was 0.796, suggesting well discriminatory capacity. Calibration curve showed favorable agreement between actual observations and predictions (Fig. 5). The high-risk threshold, standardized net benefit, and benefit ratio of the nomogram were determined by DCA (Fig. 6). The threshold probability for 3- and 5-year survival was > 0, indicating that the use of the nomogram provided more benefits than treatment/non-treatment for predicting the survival. Nomogram model also showed more benefits for predicting the 3- and 5-year OS of these patients than TNM stage. The area under the ROC curve for 1-, 3-, and 5-survival of the training group was 0.840, 0.778, and 0.871, respectively (Fig. 7A).

### Nomogram validation

The validation group was used for nomogram validation. The C-index for this group was 0.786, suggesting well discrimination. Calibration curve indicated good concordance between actual prognosis and predicted probabilities (Fig. 5). The DCA curve showed that the use of the nomogram provided more benefits than treatment/non-treatment and TNM stage for predicting the 3- and 5-year OS of all CRC patients (Fig. 6). The area under the ROC for 1-, 3-, and 5-year survival of the training group was 0.774, 0.828, and 0.803, respectively (Fig. 7). The above data suggested that the nomogram was also accurate in the validation group.

### Risk group stratification

Patients were then categorized into two subgroups based on the median of riskscore. In both training and validation sets, high-risk subgroups showed significantly worse OS (Fig. 7B, D). Moreover, the actual 5-year survival rate of the high-risk group was also lower than that of the low-risk group (80.7% vs. 60.4%, P = 3.01e-06). The score distribution and the numbers of patients in different subgroups are shown in Fig. 7.

## Discussion

Colorectal cancer is one of the most common cancers diagnosed in men and women, which remains a major cause of cancer-related death worldwide. Surgery is the main curative treatment for patients with colorectal cancer [21]. The OS of CRC patients after surgical resection varies significantly [22]. The TNM stage has currently been applied to predict the prognosis of postoperative CRC patients but with insufficient accuracy [23, 24]. Thus, it is necessary to develop a prognostic model for personalized probabilistic predictions. In the present study, a nomogram model for predicting the OS of CRC patients following surgery was constructed and validated.

The raw data of recruited patients were complete and of high quality. Univariate analysis was applied to identify prognostic variables. LASSO regression was used to select clinical features and reduce the likelihood of overfitting [25]. Over-fitting means that the established model performs too well in the training samples, resulting in poor performance in the validation data set and the test data set. Through LASSO regression and multivariate analysis, we distinguished number of positive LN, CEA levels, CA-199 levels, M stage, TNM stage, and macroscopic classification as prognostic factors.

CEA is a potential biomarker for tumor stage and the prognosis of CRC patients. Preoperative serum CEA levels were positively associated with LN invasion, vascular invasion, and perineural invasion [26]. Moreover, Hermunen et al. reported that patients with elevated postoperative CEA levels had worse OS and disease-free survival [27]. Serum CA-199 levels were positively correlated with early recurrence of CRC and negatively associated with the OS for CRC patients [28]. Also, high preoperative serum CA-199 levels could pridict worse 3-year OS and relapse-free survival [29], suggesting that CA-199 is an unfavorable prognostic factor for the survival of CRC patients. The number of positive LN is also considered a key prognostic factor for CRC patients after curative resection [30–32]. However, studies have shown that the log of the ratio of numbers of positive and negative LN is a more accurate prognostic indicator than the number of positive LN [33, 34], which therefore would be included in our upcoming studies. Macroscopic classification is another independent risk factor for recurrence of Stage II CRC [35]. Li et al. showed that macroscopic classification was an independent prognostic factor for stage I-III CRC and infiltrative CRC subtype was correlated to poor OS of stage III CRC patients [36]. Collectively, infiltrative CRC subtype is an unfavorable prognostic factor for the survival of CRC patients. The TNM staging system plays a critical role in predicting the prognosis [37]. Consistently, our study showed that TNM stage and M stage and were significantly associated with the OS of CRC patients. However, previous literature indicated that CRC patients with the same M or TNM stage had different OS and TNM stage cannot predict the prognosis of individual patients [23, 24, 38]. Here, M and TNM stage were included in the nomogram, which predicted the prognosis of each patient more accurately. Hence, inclusion of these characteristics into our nomogram was consistent with previous results.

The nomogram was constructed using characteristics selected by multivariate analysis of the training group. C-index, calibration curve, DCA, and ROC analysis were performed to evaluate nomogram performance. Then, CRC patients were categorized into high- and low-risk subgroups. For those in the high-risk subgroup, additional treatments and follow-up care may improve their prognosis. Validation is a key step in nomogram studies, which determines the generalizability of the nomogram [39]. Here, the C-indices suggesting that the nomogram model had good discriminatory capacity. The calibration curves of the training group indicated a favorable agreement between predictions and observations of 3- and 5-year outcomes, but not 1-year survival. The calibration of the validation group also suggested a favorable agreement in 3- and 5-years outcomes. These data proved the reliability and repeatability of this model for 3- and 5-year OS. Furthermore, DCA indicated that the nomogram model provided more benefits than treatment/non-treatment and TNM stage for predicting the 3- or 5-year OS of all CRC patients, indicating decent predictive and discriminatory capacity of this model. However, the prediction of 1-year survival was not accurate enough, probably due to insufficient sample size. Additionally, high-risk subgroups had significantly worse OS, suggesting satisfactory predictive performance of the nomogram.

In addition, many researchers have established nomograms for the prognosis of patients undergoing colon cancer surgery. Shuanhu Wang et al. develop and validate a prognostic nomogram for patients with resectable colon cancer, and indicate that age, race, primary site, grade, T stage, N stage, chemotherapy, and CEA level were independent predictors of OS [11]. Moreover, CEA levels (OR = 1.00, P = 0.001) was also independent predictors of OS in our study. This proves that CEA has an important effect on the prognosis of CRC patients. Y. Kanemitsu et al. develop a nomogram to predict survival of patients radical resection of colon cancer [12]. Predictors of OS were: age, gender, depth of tumour invasion, lymphatic invasion, CEA level, number of metastatic lymph nodes, number of lymph nodes examined and extent of lymphadenectomy. CEA level and number of metastatic lymph nodes were also included in our nomogram. This proves that the above variables are crucial for predicting the prognosis of patients. Chaoran Yu et al. established a nomogram model of overall survival in elderly colorectal cancer (ECRC) patients (Age ≥ 70) based on SEER database, and indicate that sex, gender, marital status, grade, AJCC TNM, metastasis and tumor size was independent predictors of OS [13]. Age and sex were independent predictors of OS in the study of Y. Kanemitsu and Chaoran Yu et al., and were included in the nomogram. And in this study, they were not included in the nomogram. This may be due to the insufficient sample size in our study. However, it is worth noting that these studies have the characteristics of long data set time span and few included variables. This raises the question of whether these nomogram can be applied to current patients. In this study, novel pathological and molecular markers, such as perineuronal infiltration, mismatch repair status, and RAS/RAF mutation status, were incorporated into the data analysis. This will help predict the survival rate of colon cancer patients more accurately.

The limitations of this work included small sample size and single-center design. Also, some prognostic factors could not be included due to the retrospective design. Data from multiple institutions will be used for external validation in future studies.

## Conclusion

A nomogram for predicting the OS of CRC patients after surgery was developed and validated, which may help clinicians predict the survival of each CRC patient and identify high-risk patients who may need more aggressive treatments.

## Data Availability

The datasets generated and analysed during the current study are not publicly available due to the protection of patient privacy but are available from the corresponding author on reasonable request.
